# Causal relationships between gut microbiome and obstructive sleep apnea: a bi-directional Mendelian randomization

**DOI:** 10.3389/fmicb.2024.1410624

**Published:** 2024-08-23

**Authors:** Liangfeng Liu, Guanwen He, Rong Yu, Bingbang Lin, Liangqing Lin, Rifu Wei, Zhongshou Zhu, Yangbin Xu

**Affiliations:** ^1^Department of Otolaryngology, Head and Neck Surgery, Ningde Municipal Hospital of Ningde Normal University, Ningde, Fujian, China; ^2^Department of Pediatrics, Jiaocheng District Maternal and Child Health Hospital, Ningde, Fujian, China; ^3^Fujian Medical University, Fuzhou, Fujian, China

**Keywords:** causal effect, gut microbiota, Mendelian randomization, obstructive sleep apnea, risk factor

## Abstract

**Background:**

Previous studies have identified a clinical association between gut microbiota and Obstructive sleep apnea (OSA), but the potential causal relationship between the two has not been determined. Therefore, we aim to utilize Mendelian randomization (MR) to investigate the potential causal effects of gut microbiota on OSA and the impact of OSA on altering the composition of gut microbiota.

**Methods:**

Bi-directional MR and replicated validation were utilized. Summary-level genetic data of gut microbiota were derived from the MiBioGen consortium and the Dutch Microbiome Project (DMP). Summary statistics of OSA were drawn from FinnGen Consortium and Million Veteran Program (MVP). Inverse-variance-weighted (IVW), weighted median, MR-Egger, Simple Mode, and Weighted Mode methods were used to evaluate the potential causal link between gut microbiota and OSA.

**Results:**

We identified potential causal associations between 23 gut microbiota and OSA. Among them, *genus Eubacterium xylanophilum group* (OR = 0.86; *p* = 0.00013), *Bifidobacterium longum* (OR = 0.90; *p* = 0.0090), *Parabacteroides merdae* (OR = 0.85; *p* = 0.00016) retained a strong negative association with OSA after the Bonferroni correction. Reverse MR analyses indicated that OSA was associated with 20 gut microbiota, among them, a strong inverse association between OSA and *genus Anaerostipes* (beta = −0.35; *p* = 0.00032) was identified after Bonferroni correction.

**Conclusion:**

Our study implicates the potential bi-directional causal effects of the gut microbiota on OSA, potentially providing new insights into the prevention and treatment of OSA through specific gut microbiota.

## 1 Introduction

Obstructive sleep apnea (OSA) is a sleep disorder characterized by repetitive episodes of complete or partial upper airway obstruction during sleep, which has emerged as a very relevant public health problem. The main symptoms of OSA are disrupted breathing, intermittent hypoxia, and frequent awakenings (Ferini-Strambi et al., 2017; Seda et al., 2021), typically accompanied by loud snoring, deteriorated sleep quality, excessive daytime sleepiness, and reduced concentration. As a highly prevalent disease, OSA significantly affects the lives of millions of people each year (Wiegand and Zwillich, 1994). It is reported that the occurrence of OSA has reached 20–30% in the adult population (Sanchez-de-la-Torre et al., 2020) and 3–5% within children (Chan et al., 2020). Nowadays, OSA brings strict challenges to both individuals and society. In addition to deteriorated life quality, patients with OSA may probably suffer from mid-term and long-term consequences, including cardiovascular, metabolic, cognitive, and cancer-related alterations (Moreno-Indias et al., 2015). What is more, sequelae of OSA will reduce work productivity and elevate the risk of motor vehicle accidents (Teran-Santos et al., 1999), which is harmful to society in the aspects of both financial and public safety. Given the ongoing trends in the obesity epidemic observed in developed and developing nations, there is an anticipation of a further increase in the global number of patients afflicted with OSA, primarily due to the strong correlation between overweight/obesity and OSA (Lam et al., 2012). However, the current diagnosis and therapy strategies for OSA are insufficient. OSA is frequently undiagnosed, while the cost resulting from undiagnosed OSA was as high as $149.6 billion in the United States. Besides, traditional treatments such as continuous positive airway pressure (Munir et al., 2023) and mandibular advancement devices are plagued by problems with adherence (Rotenberg et al., 2016), following discomfort (Dissanayake et al., 2021), and additional risks from invasive procedures (Badran et al., 2020). Therefore, it is imperative to investigate the etiology of OSA to better prevent its occurrence, make diagnoses in the early time, and explore new treatment methods for OSA.

In recent years, the relationship between microbiota composition and the pathogenesis of multiple diseases including OSA has attracted widespread attention in the academic community (Cai et al., 2021; Guo et al., 2023). In the human microbiota community, the predominant constitution is gut microbiota, which refers to the whole microbial population that colonizes the intestinal tract, including bacteria, archaea, viruses, and protozoans (Guo et al., 2023; Munir et al., 2023). The gut microbiota is involved in regulating human metabolism and immune activity and plays a crucial role in maintaining homeostasis (Musso et al., 2010; Maynard et al., 2012; Frosali et al., 2015; Fujisaka et al., 2018). Furthermore, increasing evidence suggests that the gut microbiota is associated with the onset and progression of many diseases, such as metabolic disorders, autoimmune diseases, and tumors (De Luca and Shoenfeld, 2019; Scheithauer et al., 2020; Matson et al., 2021). With the introduction of the brain-gut axis concept, there is increasing interest in the interaction between the gut microbiota and OSA. Previous studies have shown that the brain-gut axis may be a potential regulatory factor in the interaction between OSA and gut microbiota dysbiosis (Han et al., 2022). OSA may influence the gut microbiota through disrupted sleep patterns, and gut microbiota imbalance, in turn, may affect sleep patterns through changes in metabolites, creating a cycle (Munir et al., 2023). Human studies have shown that the microbiota composition differs in short sleepers compared to those with normal sleep duration. On the other hand, mice exhibit abnormal sleep patterns when the gut microbiota is depleted due to prolonged treatment with broad-spectrum antibiotics (Wang et al., 2022). These previous findings reveal clinical associations between the gut microbiota and OSA but still fail to identify the potential causality between the two. Traditional observational research may hinder the exploration of causal relationships due to its instinct bias from the reverse causality or confounders.

Large-scale cohort studies are still needed to establish the correlation between OSA and gut microbiota imbalance, as well as to determine whether treating OSA can restore the gut microbiota or if administering prebiotics and probiotics can treat OSA (Badran et al., 2020). However, these studies are costly, cumbersome, and difficult to control, which have low feasibility. Recently, Mendelian Randomization (MR) has been utilized as a novel epidemiology strategy to investigate the causal effect of certain factors on the risk of disease outcomes. According to the law of independent assortment, genetic variants will be randomly assorted to gametes during meiosis. MR analysis is a research method that uses this law as a principle to simulate randomized controlled trials (RCT) using single nucleotide polymorphisms (SNPs) as genetic instrumental variables (IVs) (Luo et al., 2023). It can eliminate confounding bias and is advantageous for separating the causal pathways of phenotypically grouped risk variables that are hard to randomize or susceptible to measurement error (Huang et al., 2023). Nowadays, MR approaches have been used to identify several risk factors for OSA, such as blood metabolites (Wu et al., 2023). Therefore, the MR method is suitable for the exploration of the causal relationships between gut microbiota and OSA. In this study, we carried out a bidirectional two-sample MR study to comprehensively assess the potential causal effects of the gut microbiota on OSA and OSA on altering the composition of gut microbiota, thus identifying specific pathogenic or protective bacterial taxa and clarifying the interactions between gut microbiota and OSA.

## 2 Methods

### 2.1 Study design

**Figure 1:**
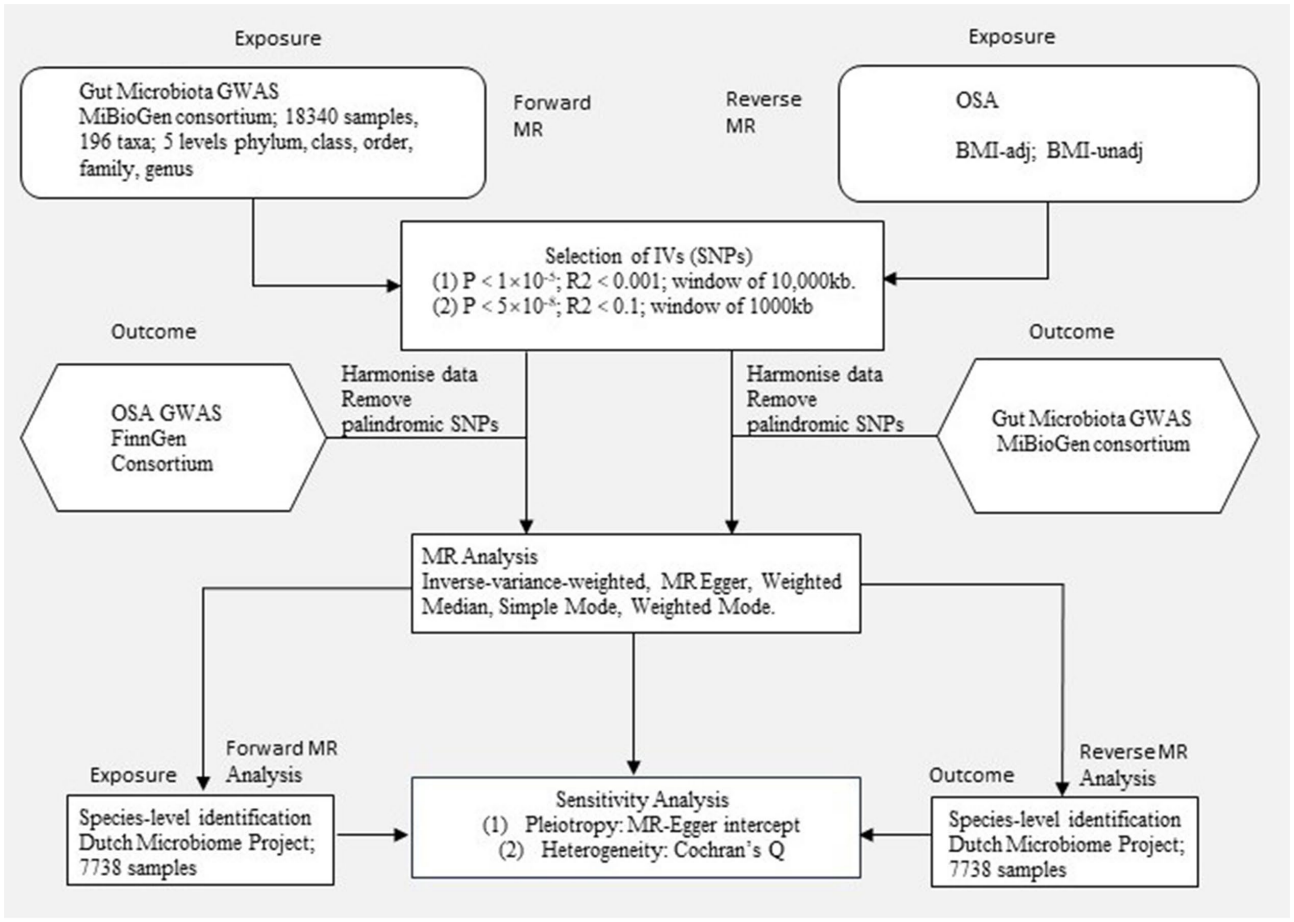
Study design. An overview of the study design. MR, Mendelian randomization; OSA, Obstructive sleep apnea; BMI-adj, OSA adjusted with body mass index; BMI-unadj, OSA unadjusted with body mass index; SNP, single nucleotide polymorphism; IVW, Inverse-variance weighted; GWAS, Genome-wide association study.

The study flow is depicted in Figure 1, illustrating the application of the two-sample MR method to evaluate the potential causal relationship between gut microbiota composition and OSA. Our study is closely adhered to the guideline of reporting MR - MR-STROBE (Supplementary file). In summary level MR analysis, SNPs are used as IVs and must satisfy three key assumptions of MR analysis (Strausz et al., 2021): (1) the relevance assumption, which requires strong correlation between IVs and the exposure of interest; (2) the independence assumption, which necessitates that IVs are not associated with confounders related to the exposure or outcome; and (3) the exclusion assumption, which mandates that IVs only affect the outcome through the exposure. Adherence to these assumptions is crucial in two-sample MR analysis to mitigate potential bias in causal estimates. To study the bi-directional causal effects between OSA and gut microbiota, we obtained human gut microbiota genetic data at the phylum to genus level from the MiBioGen, and then acquired species-level summary statistics of gut microbiota from the Dutch Microbiome Project (DMP). Summary-level OSA data is obtained from publicly available Genome-Wide Association Study (GWAS) provided by the FinnGen Consortium and Million Veteran Program (MVP). For significant estimates identified in geneus level with data from MiBioGen, we performed species level MR using data from the DMP. The potential causality of gut microbiota and OSA was calculated using statistical methods including Inverse Variance Weighted (IVW), MR Egger, Weighted Median, Simple Mode, and Weighted Mode. Among these methods, IVW is considered the primary approach. In order to reduce the risk of Type I errors, we apply Bonferroni correction to all positive results.

### 2.2 Data sources

We used a published GWAS summary statistics from FinnGen Study that contained 217,955 healthy individuals and 16,761 patients with OSA from European (Sofer et al., 2023). In this GWAS, OSA diagnosis relied on International Statistical Classification of Diseases (ICD) codes (ICD-10: G47.3, ICD-9: 3472A), which were derived from subjective symptoms, clinical examination, and sleep records (AHI ≥ 5 or respiratory event index ≥ 5).

We used another published GWAS summary statistics of OSA from the VA MVP, with *N* = 568,576. The overall OSA prevalence is 21.3%. The average age of the participants is 64 years (standard deviation of 15 years), and 91.3% of the participants are male. The analysis considered BMI and stratified the population by gender and approximate ethnicity and race, while prioritizing genetic similarity (Lopera-Maya et al., 2022). In our analysis, we used both OSA and BMI-adjusted OSA summary data.

A large-scale association study on genetic variants of the gut microbiota was conducted across 24 cohorts, involving a total of 18,340 participants from diverse populations in Canada, the United States, Israel, South Korea, Denmark, Germany, the Netherlands, Belgium, Sweden, the United Kingdom, Finland, and Denmark. The majority of the subjects (16 cohorts, *N* = 13,266) were of European ancestry, with 17 cohorts (*n* = 13,804) having participants with mean ages ranging between 50 and 62. The microbiome quantitative trait locus mapping study for each cohort focused on taxa present in more than 10% of the samples, totaling 211 taxa (131 genera, 35 families, 20 orders, 16 classes, and nine phyla). The analysis of binary trait locus mapping encompassed taxa comprising 10–90% of the included samples, resulting in 196 taxa being included in the analysis after excluding 15 taxa that could not be definitively classified and named.

For the analysis at the species level, we utilized an another GWAS dataset from the DMP, which comprised 7,738 participants. The microbiota data of these participants were subjected to quality control procedures with LifeLines (Kurilshikov et al., 2021). Among the DMP members, 58.1% were women, and their ages spanned from 8 to 84 years, with a mean age of 48.5 years.

No additional ethics approval or informed consent was required due to our study was based on public databases.

### 2.3 Instruments variables selection

In the investigation of the link between the microbiota and OSA using MR, two thresholds were applied to select the IVs. In order to ensure the genetic variations representing the microbiota trait were sufficiently strong, a locus-wide significance threshold of *p* = 1 × 10^–5^ was chosen, which is commonly used in previous microbiota MR analyses (Purcell et al., 2007). The IVs were also clustered for independence using PLINK in the two-sample MR tool (Peloso et al., 2021) and the 1,000 Genomes European data as the reference panel, with a looser cutoff of *R*^2^ < 0.001 and a clumping window of 10,000 kb.

Sofer et al. (2023) identified 24 top variants associated with OSA in multi-ethnic analysis (sex combined) in BMI-adj (OSA adjusted with BMI) and BMI-unadj (OSA unadjusted with BMI) analyses, which thorough details were provided elsewhere (Hunter-Zinck et al., 2020), and performed association analyses using PLINK v2.00a3LM (Chang et al., 2015). To ensure that the genetic variants representing OSA are sufficiently strong, they chose a genome-wide significance threshold of *p* < 1 × 10^–8^. Clumping was performed with parameters of 1,000 Kbp and *R*^2^ = 0.1 to define the top hits, using MVP multi-population genotypes as a reference panel.

### 2.4 Mendelian randomization analysis

In this study, we employed the IVW method as the primary analysis to initially assess the potential causal effects of each phenotype on OSA risk. IVW is a meta-analysis of the variant specific Wald ratios for each variant. One important assumption for IVW estimation is that the genetic variants are independent of each other (Pierce and Burgess, 2013). And the procedure of IV selection has ensured this assumption. Additionally, we conducted robustness validation using the weighted median, MR-Egger, Simple Mode, and Weighted Mode analysis. Weighted median, a method for Mendelian randomization using summary data that offers protection against invalid instruments. This approach can provide a consistent estimate of the causal effect even when up to 50% of the information contributing to the analysis comes from genetic variants that are invalid IVs. In a simulation analysis, it is shown to have better finite-sample Type 1 error rates than the inverse-variance weighted method (Bowden et al., 2016). MR-Egger can detect some violations of the standard instrumental variable assumptions, and provide an effect estimate which is not subject to these violations. The approach can used as a sensitivity analysis for assessing whether the effect estimation in a Mendelian randomization analysis is influenced by directional pleiotropic effects of the genetic variants (Bowden et al., 2015). Simple mode is a model-based assessment approach that offers pleiotropy robustness (Yang et al., 2023). Weighted Mode can obtain robust causal effect estimates for horizontal pleiotropy, it requires that the most common causal effect estimate is a consistent estimate of the true causal effect and presents less bias and lower type-I error rates than other methods under the null in many situations (Hartwig et al., 2017). The results from these methods complemented those estimated by the IVW method. In cases where the causal effects estimated by the five methods were inconsistent for a specific phenotype, a more stringent genome-wide significance threshold was applied to reselect the IVs and recalculate the causal effects (Chen et al., 2021). If the causal estimates of more than three MR methods were nominally significant, gut microbiota taxa could be considered to have potential causal effects on OSA.

As for sensitivity analysis, we assessed potential heterogeneity using Cochran’s *Q* statistics and estimated horizontal pleiotropy through the MR-Egger intercept test.

### 2.5 Statistical analysis

We applied Bonferroni correction to establish significance thresholds for the primary MR results at each taxonomic level (phylum, class, order, family, and genus). For a given feature level containing *n* bacterial taxa, the Bonferroni-corrected significance threshold was set at 0.05/n. For instance, in the case of the phylum-level MR results, with nine taxa included, the Bonferroni-corrected threshold for the *p*-value was 0.05/9 (5.56 × 10^–3^). Similarly, for the MR results at the class, order, family, and genus levels, the Bonferroni-corrected thresholds for the p-value were 3.13 × 10^–3^, 2.5 × 10^–3^, 1.56 × 10^–3^, and 4.20 × 10^–4^, respectively. Regarding Bonferroni-correction at the species level, based on its bacterial taxonomic groups, n species were identified with Bonferroni-corrected *p*-values of 0.05/n. MR results with *p*-values lower than the Bonferroni-corrected threshold were considered significant. Additionally, MR estimates with *p* < 0.05 were considered nominally significant. All *p*-values for other test reports in this study were two-tailed, and a *p* < 0.05 was considered to indicate a significant difference.

The analyses described above were primarily conducted using the Two-Sample-MR package (version 0.5.5) within the R software (version 4.0.2).

## 3 Results

### 3.1 Overview

For 196 taxa in the MiBioGen consortium, the genetic variants used as IVs for each taxon exposure ranged from 3 to 20 SNPs. For 101 species in the DMP, the genetic variants used as IVs for each species exposure ranged from 3 to 17 SNPs. Additionally, we identified 3–12 SNPs as IVs for OSA (BMI-adj and BMI-unadj) in MVP (*p* < 5 × 10^–8^). The F statistics of all retained SNPs were over 10, indicating sufficient correlation strength between IVs and exposure. See Supplementary Tables S1–S3 for the final list of retained SNPs.

### 3.2 Forward MR

**Figure 2:**
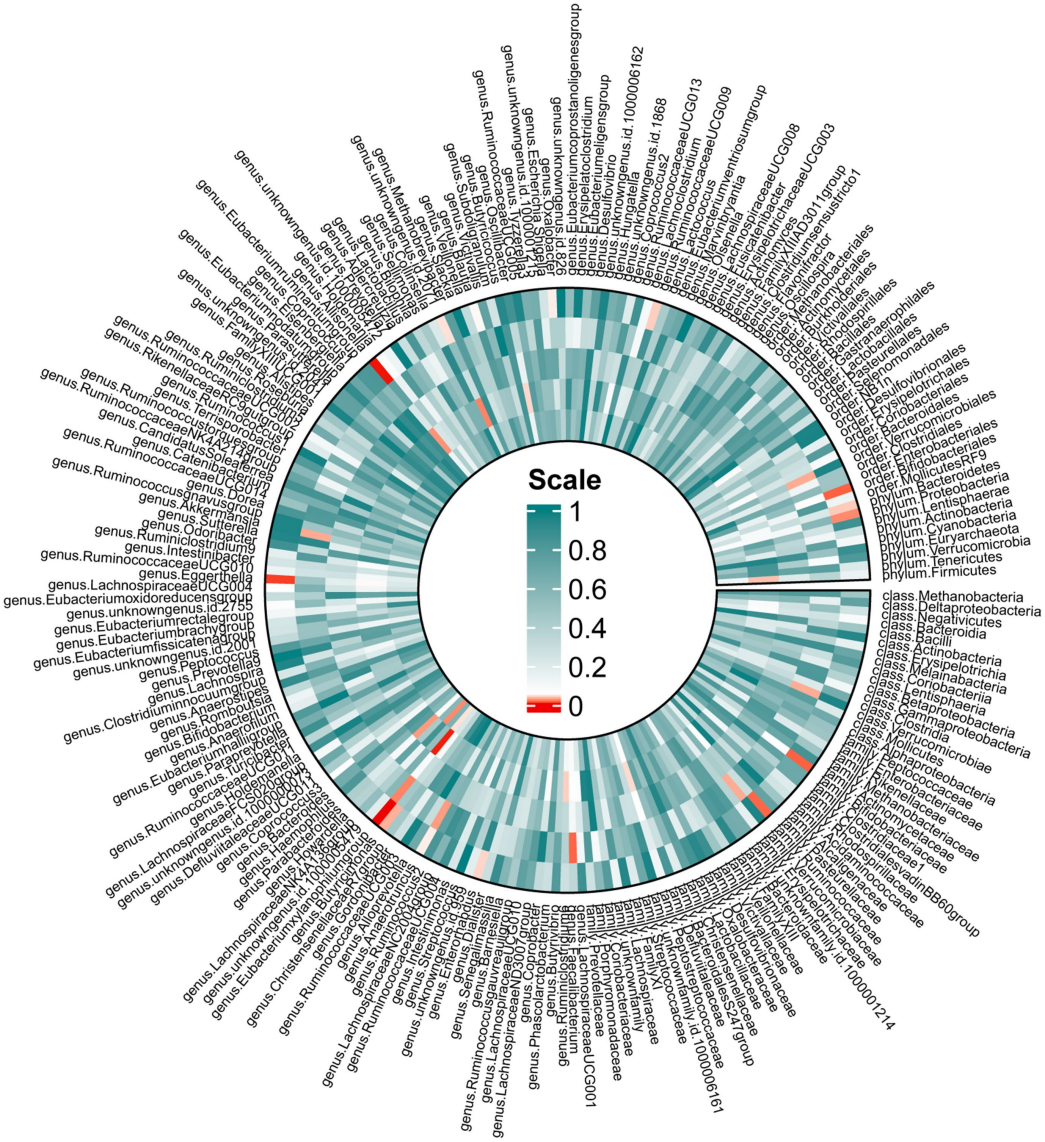
Mendelian randomization revealing causal effect from gut microbiome (MiBioGen) on OSA. From the outer to inner, cells represent *p*-values of IVW, MR-Egger, weighted median, weighted mode, simple mode. The redder, the smaller *p-*value was.

In the forward MR, we used genetic data of the gut microbiota as the exposure and genetic data of OSA from the FinnGen Consortium as the outcome (Supplementary Table S4). Based on IVW analysis, we identified 15 microbial taxa in the MiBioGen consortium that potentially had a causal relationship with OSA (Figure 2; Table 1). On the one hand, *genus Eubacterium xylanophilum group* [IVW OR = 0.86; 95% CI (0.79–0.93); *p* = 0.00013], *genus Eggerthella* [IVW OR = 0.93; 95% CI (0.88–0.98); *p* = 0.006], *family Bifidobacteriaceae* [IVW OR = 0.88; 95% CI (0.80–0.97); *p* = 0.012], *order Bifidobacteriales* [IVW OR = 0.88; 95% CI (0.80–0.97); *p* = 0.012], *family Ruminococcaceae* [IVW OR = 0.90; 95% CI (0.83–0.98); *p* = 0.013], *phylum Proteobacteria* [IVW OR = 0.91; 95% CI (0.83–0.99); *p* = 0.021], *phylum Bacteroidetes* [IVW OR = 0.91; 95% CI (0.82–0.99); *p* = 0.037], *genus Enterorhabdus* [IVW OR = 0.92; 95% CI (0.85–1.00); *p* = 0.04], *genus unknowngenus* [IVW OR = 0.93; 95% CI (0.86–1.00); *p* = 0.043], *genus Anaerotruncusshowed* [IVW OR = 0.92; 95% CI (0.84–1.0); *p* = 0.046], *genus Blautia* [IVW OR = 0.91; 95% CI (0.82–1.00); *p* = 0.048] were negatively correlated with OSA. On the other hand, *genus Allisonella* [IVW OR = 1.08; 95% CI (1.03–1.13); *p* = 0.00096], *genus Butyricimonas* [IVW OR = 1.08; 95% CI (1.01–1.16); *p* = 0.03], *genus RuminococcaceaeUCG009* [IVW OR = 1.06; 95% CI (1.0–1.13); *p* = 0.038], *genus Oxalobacter* [IVW OR = 1.046; 95% CI (1.001–1.094); *p* = 0.046] were positively correlated with OSA (Table 1). In order to further elucidate the causal relationship between gut microbiota and OSA at the species level (Supplementary Table S5), we used the positive results mentioned above as a preliminary condition and identified eight species-level microorganisms with a causal relationship with OSA in the DMP (Table 1). The results are as follows, *s_Bifidobacterium_longum* [IVW OR = 0.902; 95% CI (0.834–0.9745); *p* = 0.00903], *s_Faecalibacterium_prausnitzii* [IVW OR = 0.92297; 95% CI (0.85801–0.99284); *p* = 0.03131], *s_Desulfovibrio_piger* [IVW OR = 0.9278; 95% CI (0.8753–0.98345); *p* = 0.01168], *s_Bacteroides_salyersiae* [IVW OR = 0.96517; 95% CI (0.93429–0.99706); *p* = 0.03258], *s_Parabacteroides_merdae* [IVW OR = 0.84572; 95% CI (0.77524–0.92262); *p* = 0.00016] were related to a reduced risk of OSA (*p* < 0.05); whereas *s_Bilophila_wadsworthia* [IVW OR = 1.07819; 95% CI (1.0104–1.15052); *p* = 0.02306], *s_Bacteroides_coprocola* [IVW OR = 1.06261; 95% CI (1.01962–1.10741); *p* = 0.00395], *s_Alistipes_senegalensis* [IVW OR = 1.07075; 95% CI (1.01565–1.12883); *p* = 0.01119] were correlated with increased OSA risk (*p* < 0.05) (Table 1). Among them, *genus Eubacterium xylanophilum* group, *s_Parabacteroides merdae*, and *s_Bifidobacterium longum* were found to be significantly associated with OSA after Bonferroni-correction, while the rest were nominally significant. The weighted median analysis showed similar results in the potential causal associations between the *genus Eubacterium xylanophilum group*, *genus Allisonella*, *genus Eggerthella*, *genus unknown genus*, *s_Bacteroides coprocola*, *s_Parabacteroides merdae*, *s_Alistipes senegalensis* and OSA. We conducted an analysis on pleiotropy (Supplementary Table S6) and heterogeneity (Supplementary Table S7). Analysis of MR-Egger intercepts revealed no indication of pleiotropy (P Intercept > 0.05). According to Cochran’s Q statistic, there was no evidence of heterogeneity across instrument effects (Cochran’s QIVW > 0.05) except *family Bifidobacteriaceae*, *order Bifidobacteriales*, *s_Bifidobacterium_longum* (Table 2).

**Table 1:** Significant and nominal significant forward MR results.

Exposure	Method	*p-*value	OR	95% CI
genus.*Eubacterium xylanophilum* group	Inverse variance weighted	0.0001*	0.86	0.79–0.93
genus.Allisonella	Inverse variance weighted	0.0010	1.08	1.03–1.13
genus.Eggerthella	Inverse variance weighted	0.0060	0.93	0.88–0.98
family.Bifidobacteriaceae	Inverse variance weighted	0.0120	0.88	0.80–0.97
order.Bifidobacteriales	Inverse variance weighted	0.0120	0.88	0.80–0.97
family.Ruminococcaceae	Inverse variance weighted	0.0134	0.90	0.83–0.98
phylum.Proteobacteria	Inverse variance weighted	0.0211	0.91	0.83–0.99
genus.Butyricimonas	Inverse variance weighted	0.0305	1.08	1.01–1.16
phylum.Bacteroidetes	Inverse variance weighted	0.0369	0.91	0.82–0.99
genus.RuminococcaceaeUCG009	Inverse variance weighted	0.0378	1.06	1.00–1.13
genus.Enterorhabdus	Inverse variance weighted	0.0397	0.92	0.85–1.00
genus.unknowngenus	Inverse variance weighted	0.0426	0.93	0.86–1.00
genus.Oxalobacter	Inverse variance weighted	0.0457	1.05	1.00–1.09
genus.Anaerotruncus	Inverse variance weighted	0.0458	0.92	0.84–1.00
genus.Blautia	Inverse variance weighted	0.0485	0.91	0.82–1.00
s_Bifidobacterium_longum	Inverse variance weighted	0.0090*	0.90	0.83–0.97
s_Faecalibacterium_prausnitzii	Inverse variance weighted	0.0313	0.92	0.86–0.99
s_Bilophila_wadsworthia	Inverse variance weighted	0.0231	1.08	1.01–1.15
s_Desulfovibrio_piger	Inverse variance weighted	0.0117	0.93	0.88–0.98
s_Bacteroides_coprocola	Inverse variance weighted	0.0040	1.06	1.02–1.11
s_Bacteroides_salyersiae	Inverse variance weighted	0.0326	0.97	0.93–1.00
s_Parabacteroides_merdae	Inverse variance weighted	0.0002*	0.85	0.78–0.92
s_Alistipes_senegalensis	Inverse variance weighted	0.0112	1.07	1.02–1.13

^*^Represents that the *p*-value meets the Bonferroni-corrected significance threshold. OR, odds ratio; CI, confidence interval.

**Table 2:** Sensitivity analysis for significant and nominal significant estimates.

Outcome	Exposure	*P*-value of Egger intercept	*p*-value of Cochran’s *Q* test
OSA	genus.*Eubacterium xylanophilum* group	0.110	0.603
OSA	genus.Allisonella	0.897	0.946
OSA	genus.Eggerthella	0.893	0.933
OSA	family.Bifidobacteriaceae	0.163	0.044
OSA	order.Bifidobacteriales	0.163	0.044
OSA	family.Ruminococcaceae	0.991	0.646
OSA	phylum.Proteobacteria	0.799	0.786
OSA	genus.Butyricimonas	0.863	0.519
OSA	phylum.Bacteroidetes	0.654	0.668
OSA	genus.RuminococcaceaeUCG009	0.726	0.576
OSA	genus.Enterorhabdus	0.508	0.707
OSA	genus.unknowngenus	0.304	0.256
OSA	genus.Oxalobacter	0.650	0.769
OSA	genus.Anaerotruncus	0.074	0.328
OSA	genus.Blautia	0.299	0.240
OSA	s_Bifidobacterium_longum	0.609	0.018
OSA	s_Faecalibacterium_prausnitzii	0.747	0.779
OSA	s_Bilophila_wadsworthia	0.377	0.617
OSA	s_Desulfovibrio_piger	0.917	0.202
OSA	s_Bacteroides_coprocola	0.908	0.995
OSA	s_Bacteroides_salyersiae	0.544	0.103
OSA	s_Parabacteroides_merdae	0.486	0.448
OSA	s_Alistipes_senegalensis	0.270	0.487
genus Ruminococcaceae UCG004	BMI	0.261	0.695
genus Phascolarctobacterium	BMI	0.630	0.274
genus Paraprevotella	BMI	0.609	0.819
genus Anaerostipes	BMI	0.728	0.395
family Prevotellaceae	BMI	0.767	0.984
genus Lachnospiraceae UCG010	BMI	0.210	0.355
genus Oxalobacter	BMI	0.350	0.493
family Peptostreptococcaceae	BMI	0.965	0.104
genus Anaerostipes	unBMI	0.738	0.801
genus Methanobrevibacter	unBMI	0.844	0.423
genus Slackia	unBMI	0.804	0.851
genus *Eubacterium rectale* group	unBMI	0.126	0.664
class Methanobacteria	unBMI	0.894	0.313
family Methanobacteriaceae	unBMI	0.894	0.313
order Methanobacteriales	unBMI	0.894	0.313
genus Intestinimonas	unBMI	0.549	0.928
family Lactobacillaceae	unBMI	0.028	0.302
genus *Clostridium innocuum* group	unBMI	0.317	0.554
s_Prevotella_copri	BMI	0.824	0.826
s_Eubacterium_rectale	unBMI	0.933	0.957

### 3.3 Reverse MR

In the reverse MR (Supplementary Tables S8, S9), we found causal relationships between BMI-adj OSA and the abundance of microbiota of 2 family, 6 genus, and 1 species. *Family Prevotellaceae* [IVW beta = 0.3552; 95% CI (0.0316 to 0.6789); *p* = 0.02574], *family Peptostreptococcaceae* [IVW beta = 0.4431; 95% CI (0.0123 to 0.8740); *p* = 0.04380], *genus Ruminococcaceae UCG004* [IVW beta = 0.5083; 95% CI (0.1061 to 0.9106); *p* = 0.01325] and *genus Paraprevotella* [IVW beta = 0.5315; 95% CI (0.0644 to 0.9986); *p* = 0.02574] were positively correlated with OSA. However, *genus Phascolarctobacterium* [IVW beta = −0.5114; 95% CI (−0.9359 to − 0.0869); *p* = 0.01822]. *genus Anaerostipes* [IVW beta = −0.3384; 95% CI (−0.6448 to − 0.0321); *p* = 0.03038]. *genus Lachnospiraceae UCG010* [IVW beta = −0.3821; 95% CI (−0.7415 to − 0.0226); *p* = 0.03721]. *genus Oxalobacter* [IVW beta = −0.5927; 95% CI (−1.1687 to − 0.0168); *p* = 0.04369]. *s_Prevotella_copri* [IVW beta = −0.5861; 95% CI (−1.0892 to − 0.0830); *p* = 0.02239] was negatively correlated with OSA (Table 3). BMI-unadj OSA was causally related to the abundance of microbiota of one class, one order, two family, seven genus, and one species. To be detailed, OSA was negatively correlated with the *genus Anaerostipes* [IVW beta = −0.3539; 95% CI (−0.5466 to −0.1611); *p* = 0.000319], *genus Methanobrevibacter* [IVW beta = −0.6863; 95% CI (−1.1242 to −0.2483); *p* = 0.00213], *genus Slackia* [IVW beta = −0.4422; 95% CI (−0.7764 to −0.1080); *p* = 0.0095], *class Methanobacteria* [IVW beta = −0.5569; 95% CI (−1.0173 to −0.0966); *p* = 0.0177], *family Methanobacteriaceae* [IVW beta = −0.5569; 95% CI (−1.0173 to −0.0966); *p* = 0.0177], *order Methanobacteriales* [IVW beta = −0.5569; 95% CI (−1.0173 to −0.0966); *p* = 0.0177], *genus Intestinimonas* [IVW beta = −0.2610; 95% CI (−0.4893 to −0.0327); *p* = 0.0250], *genus Clostridium innocuum group* [IVW beta = −0.4180; 95% CI (−0.8204 to −0.0156); *p* = 0.0417], *genus Erysipelatoclostridium* [IVW beta = −0.2536; 95% CI (−0.5055 to −0.0017); *p* = 0.048456], and *s_Eubacterium_rectale* [IVW beta = −0.3969; 95% CI (−0.7157 to −0.07815); *p* = 0.01467]. On the contrary, OSA was positively correlated with the *genus Eubacterium rectale group* [IVW beta = 0.2381; 95% CI (0.0479–0.4283); *p* = 0.0141], and *family Lactobacillaceae* [IVW beta = 0.3634; 95% CI (0.03996–0.6868); *p* = 0.02765] (Table 3). The weighted median showed similar results in the potential causal association analysis of OSA with *genus Ruminococcaceae UCG004*, *genus Phascolarctobacterium*, *genus Anaerostipes, genus Lachnospiraceae UCG010*, *family Peptostreptococcaceae, genus Anaerostipes*, *genus Slackia*, *genus Eubacterium rectale group*. We conducted an analysis on pleiotropy (Supplementary Table S10) and heterogeneity (Supplementary Table S11). The MR-Egger intercept analysis indicates that, except for *family Lactobacillaceae*, all other positive results show no pleiotropy (P Intercept > 0.05). Meanwhile, Cochran’s Q statistic shows that all positive results exhibit no heterogeneity (Cochran’s QIVW > 0.05) (Table 2).

**Table 3:** Significant and nominal significant reverse MR results.

Outcome	Exposure	Method	Beta	*p*-value	95% CI
genus Ruminococcaceae UCG004	BMI	Inverse variance weighted	0.51	0.013	0.11–0.91
genus Phascolarctobacterium	BMI	Inverse variance weighted	−0.51	0.018	−0.94–0.09
genus Paraprevotella	BMI	Inverse variance weighted	0.53	0.026	0.06–1.00
genus Anaerostipes	BMI	Inverse variance weighted	−0.34	0.030	−0.64–0.03
family Prevotellaceae	BMI	Inverse variance weighted	0.36	0.031	0.03–0.68
genus Lachnospiraceae UCG010	BMI	Inverse variance weighted	−0.38	0.037	−0.74–0.02
genus Oxalobacter	BMI	Inverse variance weighted	−0.59	0.044	−1.17–0.02
family Peptostreptococcaceae	BMI	Inverse variance weighted	0.44	0.044	0.01–0.87
genus Anaerostipes	unBMI	Inverse variance weighted	−0.35	0.0003*	−0.55–0.16
genus Methanobrevibacter	unBMI	Inverse variance weighted	−0.69	0.002	−1.12–0.25
genus Slackia	unBMI	Inverse variance weighted	−0.44	0.009	−0.78–0.11
genus *Eubacterium rectale* group	unBMI	Inverse variance weighted	0.24	0.014	0.05–0.43
class Methanobacteria	unBMI	Inverse variance weighted	−0.56	0.018	−1.02–0.10
family Methanobacteriaceae	unBMI	Inverse variance weighted	−0.56	0.018	−1.02–0.10
order Methanobacteriales	unBMI	Inverse variance weighted	−0.56	0.018	−1.02–0.10
genus Intestinimonas	unBMI	Inverse variance weighted	−0.26	0.025	−0.49–0.03
family Lactobacillaceae	unBMI	Inverse variance weighted	0.36	0.028	0.04–0.69
genus *Clostridium innocuum* group	unBMI	Inverse variance weighted	−0.42	0.042	−0.82–0.02
genus Erysipelatoclostridium	unBMI	Inverse variance weighted	−0.25	0.048	−0.51–0.00
s_Prevotella_copri	BMI	Inverse variance weighted	−0.59	0.022	−1.09–0.08
s_Eubacterium_rectale	unBMI	Inverse variance weighted	−0.40	0.015	−0.72–0.08

^*^Represents that the *p*-value meets the Bonferroni-corrected significance threshold. OR, odds ratio; CI, confidence interval.

## 4 Discussion

OSA is a widespread condition that significantly impacts individuals’ daily lives, posing risks to personal health and societal safety. Besides, recent research underscores the role of gut microbiota in the pathogenesis of various diseases. Meanwhile, the composition of gut microbiota is also suggested to be altered by several diseases, which may in turn influence the development of other conditions. Thus, identifying the bidirectional connections between gut microbiota and OSA is valuable for the management of both OSA and systemic conditions related to gut microbiota. Although some observational studies discover clinical relationships between OSA and gut microbiota, it is still unknown whether these links are potentially causal. In addition, the instinctive drawbacks of traditional observational research including reverse causality and bias from confounder factors lead to the failure to deduce the causal effects of risk factors on certain diseases. To address these issues, in this study, we utilized genetic variations as a proxy to reveal the causal relationship between gut microbiota and OSA. To our knowledge, this is the first MR study to elucidate their bidirectional causal relationships at the species level. To strengthen the reliability of the results, we utilized two gut microbiota datasets and two OSA datasets that were independent of each other for MR analyses. In the forward MR analysis, we first identified gut microbiota at the phylum, order, family, and genus levels associated with OSA. We found that an increase in the abundance of gut microbiota at six genus levels, two family levels, two phylum levels, and one order level is associated with a reduced risk of OSA, while an increase in the abundance of gut microbiota at four genus levels is associated with an increased risk of OSA. Subsequently, we determined the causal relationship between species-level gut microbiota and OSA. Building on the aforementioned results, we found that an increase in the abundance of five gut bacterial taxa is associated with a reduced risk of OSA, whereas an increase in the abundance of 3 gut bacterial taxa is associated with an increased risk of OSA. Notably, after Bonferroni-correction, we discovered a significant causal relationship between the *genus Eubacterium xylanophilum group*, *s_Parabacteroides_merdae*, and *s_Bifidobacterium_longum* and the risk of OSA.

The mechanism of how the *Parabacteroides merdae* and *Bifidobacterium longum* affect OSA may be associated with obesity. Obesity is demonstrated to be a risk factor for OSA. Among patients already diagnosed with OSA, a 10% weight gain predicted an approximately 32% increase in the AHI, while a 10% weight loss predicted a 26% decrease in AHI (Murugan and Sharma, 2008). In a 4-year follow-up cohort study, it was found that among initially non-OSA participants, the risk of developing OSA increased sixfold after a 10% weight gain (Murugan and Sharma, 2008). Obesity can lead to increased pressure on the chest and diaphragm muscles, making it harder for the respiratory muscles to work. In an animal experiment, *Parabacteroides merdae* reduced the weight gain induced by a high-fat diet in mice by 27% (Qiao et al., 2022). *Parabacteroides merdae* is positively correlated with the levels of short-chain fatty acids (SCFAs) in the cecum, including acetic acid, propionic acid, and butyric acid (Ridaura et al., 2013), which can inhibit weight gain by modulating food intake, physical activity, heart rate, and oxygen assumptions (Yamashita et al., 2009; Lin et al., 2012). What is more, it was found that the feces of mice treated with *Parabacteroides merdae* contained branched short-chain fatty acids (BSCFAs) derived entirely from branch chain amino acids (BCAAs) (Leucine, isoleucine, and valine) (Qiao et al., 2022), demonstrating the ability of *Parabacteroides merdae* to regulate the degradation of BCAAs in the gut (Qiao et al., 2022). Another experiment showed that obesity-related bacteria exhibited higher BCAAs synthesis rates and lower BCAAs degradation rates (Ridaura et al., 2013). Compared to children without OSA, children with OSA have significantly higher levels of BCAAs (Barcelo et al., 2017), which is in line with our results. In terms of *Bifidobacterium longum* strains, an animal experiment found that levels of SCFAs such as acetate, propionate, and butyrate in the feces increased after oral administration of *Bifidobacterium longum* (Gao et al., 2023). The study by Rahman et al. (2021) demonstrated that supplementation with *Bifidobacterium longum* subspecies infantis YB0411 significantly reduced body weight and fat accumulation in high-fat diet-induced obese mice. The positive anti-obesity effect of *Bifidobacterium longum* APC1472 strain in high-fat diet-induced obese mice, as well as the partial transformation of these positive effects of *Bifidobacterium longum* APC1472 supplementation in other healthy overweight and obese individuals (Schellekens et al., 2021). Likewise, *Bifidobacterium longum* subspecies infantis produces SCFAs through the metabolism of Human Milk Oligosaccharides (Chichlowski et al., 2020). Therefore, *Parabacteroides_merdae* and *Bifidobacterium_longum* may inhibit weight gain and reduce the risk of developing OSA.

In our research, we have also identified that the *genus Eubacterium xylanophilum group* is a protective factor against OSA. This genus is significantly associated with the synthesis of secondary bile acids (BAs) (Liu et al., 2022). BA metabolites have been shown to influence sleep regulatory centers and circadian rhythms (Yang and Zhang, 2020), thereby impacting human sleep quality and health. Additionally, animal experiments conducted by Ferrell and Chiang (2015) demonstrated that even short-term circadian disruption (no more than 5 days) substantially altered the expression of hepatic clock genes and BA metabolism. Furthermore, Kanemitsu et al. (2017) found that specific BAs block the activation of circadian transcription factors and the nuclear receptor peroxisome proliferator-activated receptor. It has been observed in animal models that chronic intermittent hypoxia disrupts BA metabolism (Zhang et al., 2022). On the other hand, the *genus Eubacterium xylanophilum* ferments complex phytochemicals to produce SCFAs including butyrate (Duncan et al., 2016). Butyrate has been found to be antiobesogenic in human studies (Chakraborti, 2015). The *genus Eubacterium xylanophilum group* is negatively associated with the energy-adjusted dietary inflammatory index score and is correlated with higher visceral adipose tissue (Lozano et al., 2022).

Many previous studies have shown changes in the gut microbiota of OSA patients (Tang et al., 2022; Wang et al., 2022; Zhang et al., 2022). So, we also conducted a reverse MR analysis to explore the changes in the microbiota of OSA patients. The reverse MR analysis showed that OSA was associated with 19 microbial taxa (eight BMI-adj, 11 BMI-unadj) and two microbial species (one BMI-adj, one BMI-unadj). OSA was significantly negatively correlated with the genus Anaerostipes. Further comparison of the results of forward and reverse MR analyses revealed that OSA had a self-limiting effect on the *genus Oxalobacter*. Li et al. (2023) used a prospective case–control study to examine the fecal microbiota composition of 48 subjects (by 16S rDNA gene amplification and sequencing) and found that the microbiota of patients with severe OSA was decreased with *Anaerostipes*. This is consistent with our research findings. OSA have been well described, such as circulating blood D-lactic acid (D-LA) protein are increased (Barcelo et al., 2016). D-LA is the product of the fermentation of gastrointestinal bacteria and serves as an indicator of bacterial translocation, intestinal injury, and intestinal permeability (Clausen et al., 1991; Heizati et al., 2017). The levels of D-LA are negatively correlated with *Anaerostipes* (Li et al., 2023). Dysfunction of the intestinal barrier function is associated with bacterial dysbiosis in OSA.

The gut microbiome is primarily constituted of bacteria, but it also includes archaea, viruses, fungi, and protists. Most research has focused on bacteria, while other microbiome such as viruses, archaea, fungi, and protists are often overlooked. The composition of the neglected microbial community differs not only in their average estimated physical size but also in their absolute abundance in the gut. They contribute significantly to general gut metabolism, gut homeostasis, and provide protection from pathogenic infections. This study primarily elucidates the causal relationship between gut bacteria and OSA, but archaea, viruses, fungi, protists, and their metabolites may also exert certain influences. To fully understand the interactions between the gut microbiome and OSA, future research needs to be more inclusive, addressing multiple microbial components simultaneously.

This is the first two-sample MR method to study the association between gut microbiota and OSA, which has the following advantages. Firstly, according to Mendel’s law of inheritance, alleles are randomly allocated in offspring, similar to randomization in a randomized controlled trial (Zheng et al., 2017). In addition, genotypes are fixed at conception and are not influenced by the disease (Zheng et al., 2017). Therefore, causal inference is less likely to be influenced by reverse causation and confounding factors. Secondly, two-sample MR is based on publicly available large-scale GWAS summary-level data, requiring no additional experimental costs. Thirdly, potential causal associations determined by the IVW method may provide candidate bacterial taxa for future functional studies on the mechanisms underlying the association between gut microbiota and OSA. This study has some limitations. Firstly, in the forward MR analysis, the focus was primarily on populations of European ancestry, which may limit the generalizability of the results to other populations. In the reverse MR analysis, although the majority of individuals were of European and North American ancestry, the inevitable inclusion of mixed-race individuals may introduce bias in the results. Secondly, this MR analysis of the association between gut microbiota and OSA does not explain its mechanisms. Thirdly, in our study, we utilized the summary statistics published and we could not adjust the rules for participate and exclusion. Therefore, infection and other disease might induce bias to our analysis. Finally, as the instrumental variables were derived from GWAS meta-analyses, we were unable to explore stratification effects and other non-linear relationships. While observational studies have not yet concluded whether the impact of gut microbiota on OSA risk is linear, the possibility of non-linear models cannot be ruled out. In the future, it is anticipated that individual-level GWAS data will be used for non-linear MR studies (Staley and Burgess, 2017), to further explore the non-linear relationship between gut microbiota and OSA. Despite these potential limitations, we confirmed the robustness of our causal estimates through a series of sensitivity analyses, indicating that this study accurately reflects the strong association between gut microbiota and the risk of OSA.

## 5 Conclusion

In this study, we utilized bidirectional MR to reveal the causal relationship between gut microbiota and OSA. We found that an increase in the abundance of gut microbiota at six genus levels, two family levels, two phylum levels, and one order level is associated with a reduced risk of OSA, while an increase in the abundance of gut microbiota at 4 genus levels is associated with an increased risk of OSA. Subsequently, we found that an increase in the abundance of five gut bacterial taxa is associated with a reduced risk of OSA, whereas an increase in the abundance of three gut bacterial taxa is associated with an increased risk of OSA. Our study implicates the potential causal effects of the gut microbiota on OSA and OSA on altering the composition of gut microbiota, potentially providing new insights into the prevention and treatment of OSA through specific gut microbiota.

## Data availability statement

The original contributions presented in the study are included in the article/Supplementary material, further inquiries can be directed to the corresponding authors.

## Author contributions

LFL: Writing – original draft, Formal analysis, Data curation, Conceptualization. GH: Writing – original draft, Formal analysis, Data curation, Conceptualization. RY: Writing – original draft, Formal analysis, Data curation. BL: Writing – review & editing, Formal analysis, Data curation. LQL: Writing – review & editing, Formal analysis, Data curation. RW: Writing – review & editing, Supervision, Formal analysis, Data curation. ZZ: Writing – review & editing, Supervision. YX: Writing – review & editing, Supervision.
